# High survivin expression as a risk factor in patients with anal carcinoma treated with concurrent chemoradiotherapy

**DOI:** 10.1186/1748-717X-7-88

**Published:** 2012-06-14

**Authors:** Ingeborg Fraunholz, Claus Rödel, Luitpold Distel, Marget Rave-Fränk, Daniela Kohler, Stefan Falk, Franz Rödel

**Affiliations:** 1 Department of Radiotherapy and Oncology, Johann Wolfgang Goethe-University, Theodor-Stern-Kai 7, 60590, Frankfurt am Main, Germany; 2 Department of Radiation Oncology, Friedrich-Alexander-University, Universitäts-straße 27, 91054, Erlangen, Germany; 3 Department of Radiation Oncology, Johann August-University, Robert-Koch-Straße 40, 37075, Göttingen, Germany; 4 Pathology Associates, Ginnheimer Landstraße 94, 60483, Frankfurt/Main, Germany

**Keywords:** Survivin, Anal cancer, Molecular marker, Concurrent chemoradiotherapy

## Abstract

**Purpose:**

To investigate the prognostic value of survivin expression in pretreatment specimens from patients with anal cancer treated with concurrent 5-FU and mitomycin C-based chemoradiation (CRT).

**Material and methods:**

Immunohistochemical staining for survivin was performed in pretreatment biopsies of 62 patients with anal carcinoma. Survivin expression was correlated with clinical and histopathological characteristics as well as local failure free- (LFFS), distant metastases free- (DMFS), cancer specific- (CSS), and overall survival (OS).

**Results:**

Survivin staining intensity was weak in 10%, intermediate in 48% and intense in 42% of the patients. No association between survivin expression and clinicopathologic factors (tumor stage, age and HIV status) could be shown. In univariate analysis, the level of survivin staining was significantly correlated with DMFS (low survivin vs. high survivin: 94% vs. 74%, *p* = 0.04). T-stage, N-stage and the tumor grading were significantly associated with OS and CSS and with DMFS and LFFS, respectively. In multivariate analysis, survivin was confirmed as independent prognostic parameter for DMFS (RR, 0.04; *p* = 0.02) and for OS (RR, 0.27; *p* = 0.04).

**Conclusion:**

Our results demonstrated that the level of pretreatment survivin is correlated with the clinical outcome in patients with anal carcinoma treated with concurrent CRT. Further studies are warranted to elucidate the complex role of survivin for the oncologic treatment and to exploit the protein as a therapeutic target in combined modality treatment of anal cancer.

## Background

Survivin, the smallest and structurally unique member of the inhibitor of apoptosis protein family (IAP) [1] plays a prominent role within tumor biology [2]. As a prime example of a nodal cancer protein, it is involved in the regulation of a multitude of cellular networks, including tumor cell proliferation, apoptosis and response to unfavorable environmental conditions [3]. While it is highly expressed during fetal development and is down regulated in most terminally differentiated normal tissues, the protein is found to be re-expressed in virtually every human malignancy examined so far [4,5]. In line with that, survivin has been recognized as a suitable prognostic and predictive marker for tumor onset, enhanced proliferative index, more aggressive tumor behavior and strongly correlates with a higher likelihood of tumor recurrence and impaired disease free- and overall survival rates [4,6]. Moreover, a correlation of elevated survivin expression with increased risk of recurrences, lymph node metastases, and shorter survival was shown beside others, in non-small cell lung cancer (NSCLC), T1 bladder carcinoma [7], rectal adenocarcinoma [8] and locally advanced prostate cancer [9] treated with radiation therapy or chemoradiation. Due to its universal over-expression and unique biological properties, survivin further displays a validated molecular target for cancer drug development [10,11]. A variety of preclinical studies have demonstrated that targeting the protein using RNA-interference, dominant negative mutants, antisense oligonucleotides and small molecule repressors sensitized tumor cells towards chemotherapy and irradiation and reduced tumor growth potential [6,12]. In addition, the translation of the preclinical findings into clinical practice is currently under way as a variety of survivin antagonists entered clinical phase I/II trials [13,14].

In anal cancer, the prognostic value of apoptosis associated proteins has only been evaluated in a few studies on a restricted number of patients [15-19] showing that expression of Bcl-2, M30, p53, and nuclear factor κB (NF-κB) may be an independent predictor for disease free survival (DFS) and local control (LC), respectively. By contrast, a prognostic or predictive impact of survivin expression has not yet been investigated. Thus, the objective of the present study was to evaluate the expression of survivin in pretreatment biopsy specimen of patients with anal carcinoma treated with concurrent CRT and to correlate its immunoreactive score with clinicopathologic characteristics and clinical outcome.

## Patients and methods

### Patients

62 patients, uniformly treated with definitive CRT for anal cancer in the Department of Radiotherapy and Oncology of the University Hospital of Frankfurt am Main and at the Department of Radiation Therapy of the University Hospital of Erlangen, who had available biopsy tissues and provided informed consent, were included in this study. Eligibility criteria were histological proof of anal canal carcinoma (squamous cell or basaloid or cloacogenic subtype) and curative intent of 5-FU and Mitomycin C-based CRT. Pretreatment evaluation consisted of physical and rectal-digital examination, proctoscopy with biopsy, CT/MRI of the abdomen and pelvis, chest x-ray, serum chemistry, and complete blood count in all patients. Patients were staged according to the system adopted by the Union International Contre le Cancer (UICC) and the American Joint Committee on Cancer.

### Treatment modalities and follow-up

3-D conformal radiation therapy was performed using either a 6- or 25-MV photon beam linear accelerator with individual field arrangement. The target volume included the primary tumor and the mesorectal, inguinal and internal iliac lymph nodes. The patients were treated with a median dose of 50.4 Gy (range, 41.4–60 Gy) with daily fractions of 1.8–2 Gy. A brachytherapy-boost with a median dose of 14 Gy (range, 12–22 Gy) was applied in 4 patients. 27 patients received an external boost to the primary tumor and/or enlarged lymph nodes of 5.4 Gy (range, 1.8–10.8 Gy). The anal boost volume was defined as anal gross tumor plus a safety margin of 2 cm in each direction. The dose specification was based on ICRU Report 50 recommendations. Concurrent chemotherapy consisted of two cycles of 5-FU (1.000 mg/m^2^/24 hours) as 4 or 5-day continuous infusion in the first and fifth week of radiotherapy; mitomycin C was administered as intravenous bolus on day one of each cycle. During the treatment, patients were evaluated weekly to assess acute toxicity and compliance with the chemoradiation. Initial treatment response was assessed by rectal-digital examination and proctoscopy, with biopsies taken in case of suspicious residual tumor, 6–8 weeks after completion of CRT. The lymph node status was documented by pelvic CT/MRI-scan. Patients were scheduled for follow-up examinations, including history, physical and rectal-digital examination, and proctoscopy with biopsy in case of any suspicious finding, every 3 months for 2 years, followed every 6 months subsequently. Local control was defined as control of the anal primary tumor while nodal failure was considered as locoregional relapse. The median follow up time was 68 months (range 9–246).

### Immunohistochemical detection of survivin

Immunohistochemical staining of survivin was performed on anal cancer biopsies by a horseradish-peroxidase technique using a DAKO Autostainer plus (DAKO, Hamburg, Germany) according to the manufacturer’s recommendation. For staining purpose, paraffin embedded tissue either mounted on microscope slides (Star Frost, Engelbrecht, Germany) or tissue microarrays as described in [20] were pretreated for 20 min with an Epitope Retrieval Solution (Dako). Next, slides were subjected to an automatic staining procedure with standardized H_2_0_2_ (10%) treatment (10 min) and antibody (AF886, R&D Systems, Wiesbaden, Germany) incubation (20 min, 1:750). Next, dextran polymer conjugated horseradish-peroxidase and 3,3’-diaminobenzidine (DAB) chromogen was used for visualization of the epitope-antibody reaction product and hematoxylin (37%) for counterstaining. Negative control slides in the abcense of primary antibodies and positive controls with formalin-fixed, paraffin-embedded cancer specimens of known survivin reactivity were included for each staining.

### Scoring of survivin expression

Survivin immunoreactivity was analyzed considering both the percentage of positive cells and the intensity of staining. The staining intensity was scored as: 1+ (weak), 2+ (moderate) and 3+ (intense). The fraction of tumor cells with survivin positivity was assigned to: 1 (0–25%), 2 (26–50%), 3 (51–75%) and 4 (> 75%). To minimize interobserver variability, scoring was performed by two independent investigators without knowledge of the clinicopathological data. In discrepant cases, a final decision was made based on consensus by the investigators and, if necessary, a recount of the labeled cells was done. The percentage of positive tumor cells and the staining intensity were then multiplied to produce an individual labeling score for each case, ranging from 0 (no positive tumor cells) to 12 (> 75% of tumor cells with intense staining). A weighted score ≤ 8 was defined as “low” survivin expression and a weighted score of > 8 as “high” survivin expression. This dichotomous variable served for correlation of survivin expression with clinicopathological parameters and survival.

### Statistical analysis

Mean values are indicated with standard deviation. Differences between groups on continuous variables were tested using the Mann–Whitney test. Fisher’s exact test was used to test differences between groups on categorical variables. Survival and time to recurrence were calculated from the date of CRT-beginning to the day of death and recurrences, respectively, or the date of the last follow-up. Disease free survival (DFS) was defined as the time between the start of CRT and tumor relapse (locoregional recurrence and/or distant metastases) or death due to non-tumor related causes. Local failure free survival (LFFS) was defined as the time from the start of CRT to the first local tumor detection after CRT (i.e. non-complete response), local tumor recurrence after complete response or death from any cause. Survival curves were plotted according to the Kaplan-Meier method using SPSS 15.0 for Windows. Survival comparison between groups used the log-rank (Mantel-Cox) test.

The multivariate analysis was carried out with the Cox proportional hazard models.

## Results

### Survivin immunostaining on pretreatment biopsies

**Figure 1:**
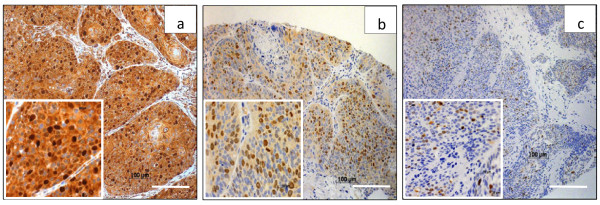
**Examples of anal cancer biopsies with intense (a), intermediate (b) and weak (c) immunohistochemical staining of survivin in both, cytoplasm and nucleus of tumor cells.** Original magnification x 100 (a,b,c) and x 400 (inserts), scale bar: 100 μm.

The intensity of survivin staining was usually homogeneous within a tumor specimen tested, but varied considerably among individual tumors. In Figure 1, representative examples of intense (>75 positive tumor cells), intermediate (25–75%) and weak (≤ 25%) survivin immunoreactivity are illustrated. The distribution of the staining characteristics and labeling scores is presented in Table 1. As a dichotomous variable, survivin expression was defined as “low” (weighted score ≤ 8) in 36 patients (58%) and “high” (weighted score > 8) in 26 patients (42%). As shown in Table 2, no significant relationship was found for the dichotomized variable and clinicopathologic parameters such as T-stage, N-stage, age and HIV status.

**Table 1:** Results of immunohistochemistry

	**Patients**
	**n (%)**
**Percentage of immunopositive cells:**	
0–25%	1 (2)
26–50%	6 (10)
51–75%	10 (16)
76–100%	45 (72)
**Staining intensity:**	
1+	6 (10)
2+	30 (48)
3+	26 (42)
**Individual labeling score:**	
1–3	6 (10)
4–8	30 (48)
9–12	26 (42)
**Dichotomized labeling score:**	
≤ 8	36 (58)
>8	26 (42)

**Table 2:** Patients characteristics

	**No. of patients**	low survivin*	high survivin**	**p-value**
		**n (%)**	**n (%)**	
**Age**				
≤ 56 years	31	19 (53)	12 (46)	0.79
> 56 years	31	17 (47)	14 (54)	
**Sex**				
Male	26	15 (42)	11 (42)	1.0
Female	36	21 (58)	15 (58)	
**HIV-status**				
Positive	15	7 (19)	8 (31)	0.37
Negative	47	29 (81)	18 (69)	
**T-stage**				
T1/2	44	26 (72)	18 (62)	0.78
T3/4	17	9 (25)	8 (31)	
Tx	1	1 (3)	0 (0)	
**N-stage**				
N0	37	18 (50)	19 (73)	0.18
N+	23	16 (44)	7 (27)	
Nx	2	2 (6)	0 (0)	
**Grading**				
G1/2	47	27 (75)	20 (77)	0.75
G3	12	6 (17)	6 (23)	
Gx	3	3 (8)	(0)	

Abbreviations: HIV = human immunodeficiency virus.

*survivin - weighted score ≤ 8; **survivin - weighted score > 8.

### Survivin and treatment response

After CRT, a clinical complete response was diagnosed in 55 patients (low survivin vs. high survivin: 89% vs. 88%, *p* = 1.0). Within these patients, a local recurrence occurred in 6 patients (16% vs. 4%, *p* = 0.38). Local control was achieved in 49 patients (75% vs. 86%, *p* = 0.53). Distant metastases were evident in 8 patients (6% vs. 23%, *p* = 0.05). Within the 15 patients who have died (19% vs. 31%, *p* = 0.37), 9 patients died from anal cancer (12% vs. 26%, *p* = 0.47), two patients died from intercurrent disease and 4 patients from unknown causes.

**Figure 2:**
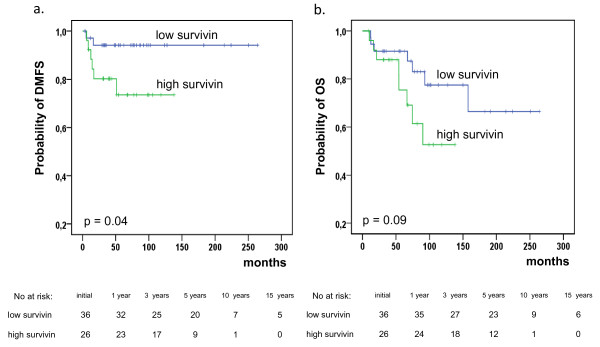
Distant metastases free survival (DMFS) (a) and Overall survival (OS) (b) according to pretreatment survivin expression (low survivin: weighted score ≤ 8; high survivin: weighted score > 8).

As displayed in Figure 2, patients with low survivin expression had a significantly superior DMFS (94% vs. 74%, *p* = 0.04). OS was also superior, but without significance (92% vs. 75%, *p* = 0.09). In univariate analysis, the T-stage, N-stage and the tumor grading were also significantly associated with OS and CSS and with DMFS and LFFS, respectively (Table 3). Factors significantly influencing the clinical endpoints such as survivin expression, T-stage, N-stage and tumor grading as well as patients’ gender were included in multivariate analysis. In the Cox models, survivin was confirmed as an independent prognostic parameter for DMFS (RR, 0.04; *p* = 0.02) and for OS (RR, 0.27; *p* = 0.04). As depicted in Table 4, T-stage was also an independent prognostic factors for DMFS (RR, 1.88; *p* = 0.02).

**Table 3:** Univariate analysis for prognostic factors influencing 5 year-survival

	**OS**	**p-value**	**CSS**	**p-value**	**DMFS**	**p-value**	**LFFS**	**p-value**
	**(%)**		**(%)**		**(%)**		**(%)**	
**Age**								
≤ 56 years	84	0.17	84	0.75	83	0.47	81	0.46
> 56 years	87		92		88		82	
**Sex**								
Male	75	0.41	80	0.30	80	0.20	72	0.08
Female	93		93		89		89	
**HIV-status**								
Positive	73	0.27	73	0.07	72	0.08	73	0.13
Negative	90		93		90		84	
**T-stage**								
T1/2	93	**0.01**	93	**<0.01**	90	**0.02**	88	**0.02**
T3/4	67		76		72		64	
**N-stage**								
N0	93	**<0.01**	93	**<0.01**	90	0.19	100	**<0.01**
N+	73		78		81		55	
**Grading**								
G1/2	91	**<0.01**	94	**0.04**	91	0.08	84	0.14
G3	64		64		65		75	
**Survivin**								
low*	92	0.09	92	0.29	94	**0.04**	80	0.56
high**	75		82		74		85	

Abbreviations: OS = overall survival; CSS = cancer specific survival; DMFS = distant metastases-free survival; LFFS = local failure-free survival; HIV = human immunodeficiency virus. *Survivin - weighted score ≤ 8; **survivin - weighted score > 8.

**Table 4:** Multivariate analysis for distant metastases-free survival (DMFS) and overall survival (OS)

	**RR**	**DMFS**		**RR**	**OS**	
		**95% CI**	**p-value**		**95% CI**	**p-value**
**Sex**						
Male/female	1.33	0.14–12.94	0.81	1.07	0.27–4.21	0.92
**HIV-status**						
Positive/negative	0.24	0.03–2.08	0.20	0.43	0.09–2.00	0.28
**T-stage**						
T1–2/T3–4	1.88	1.10–3.21	**0.02**	1.31	0.96–1.80	0.08
**N-stage**						
N0/N+	1.35	0.89–2.03	0.15	1.18	0.95–1.48	0.14
**Grading**						
G1-2/G3	1.62	0.91–2.89	0.10	1.15	0.79–1.67	0.47
**Survivin**						
Low*/high**	0.04	0.003–0.59	**0.02**	0.27	0.08–0.92	**0.04**

Abbreviations: DMFS = distant metastases-free survival; OS = overall survival; RR = relative risk; CI = confidence interval; HIV = human immunodeficiency virus. *Survivin - weighted score ≤ 8; **survivin - weighted score > 8.

## Discussion

Despite respectable treatment response in non-metastatic anal carcinoma with OS rates of 60–75%, only a small amount of patients (10%) with distant metastases will survive more than two years from the time of diagnosis [21]. Although clinical and pathological factors like tumor size and extent and nodal involvement have been regarded to be of prognostic significance, biomarkers have not yet been consistently be proven to predict tumor response [22]. Whereas some studies reported on a prognostic value of TP53, p21 and cyclin A, other investigations did not [23]. Thus, extended insights into individual tumor characteristics and the development of novel molecular indicators are seriously needed.

Deregulation of apoptosis is a pathogenic factor of many human diseases, and the ability to circumvent or evade apoptotic cell death is a critical hallmark of tumor cells [24]. Since deregulation in cell death pathways, for example by an elevated expression of anti-apoptotic molecules, may result in resistance to conventional treatment regimens including chemo and radiation therapy, these markers may be a novel class of molecular predictors. In the present study, we investigated the predictive and prognostic relevance of the apoptosis inhibitor survivin in biopsies derived from patients with anal carcinoma treated with definitive CRT. By using histochemical evaluation and scoring we demonstrated that pretreatment survivin expression is correlated with the clinical outcome-namely DMFS. These findings are in line with a variety of studies showing that a high survivin expression is correlated with increased risk of tumor recurrences, lymph node metastases and lower survival after definitive radiotherapy or chemoradiation in esophageal [25], cervical [26], lung [27], early (T1/T2) prostate [9], and in nasal/paranasal sinus cancer [28]. More recent data further indicate that intratumoral survivin expression significantly decreased during neoadjuvant chemoradiation in esophageal and rectal cancer [29,30]. A failure in radiation provoked down-regulation, by contrast, was significantly associated with shortened overall survival and an increased likelihood to develop distant metastases. These results support the concept that measuring survivin expression may be suitable to predict individual metastatic behavior. To strengthen this hypothesis, an intermolecular cooperation between survivin and its family partner X-linked IAP (XIAP) has recently been reported to stimulate an invasive behavior and promote tumor cell metastases. Thus, both proteins are considered to modulate metastasis progression, possibly orchestrating a cellular network of transcription factor NF-κB dependent expression of fibronectin, β-integrin signaling and activation of the cell motility kinases focal adhesion molecule (FAK) or matrix metalloproteinases [31,32].

Due to its differential expression in cancerous and normal tissue and its requirement for regulating apoptosis, maintaining cancer cell viability and to modulate therapy response, survivin is supposed to be a suitable target for a molecular tumor therapy [10,11]. Indeed a variety of preclinical studies have convincingly demonstrated that survivin is a radiation resistance factor [33-35]. Moreover, attenuation of the protein by antisense oligonucleotides (ASO), small interfering RNAs (siRNAs), small molecule transcriptional inhibitors and peptidomimetics impacted on apoptosis or clonogenic survival, radiosensitized glioblastoma [33], colorectal carcinoma [36], non-small cell lung cancer cells [37], and reduced tumor growth in xenograft models [6,12]. The translation of these preclinical findings to the clinical practice is currently under way with a number of phase I/II trials targeting survivin by the use of ASO (LY2181308) and small molecule inhibitors (YM155) in progress [13,14]. However, first results indicate modest activity as single agents [38,39], but it is anticipated that when given combined with conventional chemotherapeutic drugs or irradiation these agents may exhibit enhanced individual success. Thus it is tempting to speculate, that combined anti survivin and CRT therapy in anal carcinoma will emerge as a novel treatment option in patients overexpressing the protein.

To the best of our knowledge, this is the first report evaluating survivin expression in anal carcinoma undergoing definitive CRT based on a well documented patient cohort and tumor material available. Although our investigation has some limitations due to restricted number of patients, low statistical power, and potential cut-point bias we consider the results as representative for a larger group of patients. Nevertheless, a prospective validation of the impact of survivin expression in anal carcinoma has to be performed on the basis of a clinical trial including a standardized protocol of immunohistochemical evaluation of the protein in pretreatment specimens.

In conclusion, we have shown that a histochemical evaluation of survivin expression in pretreatment biopsies of patients with anal carcinoma provides an easy accessible molecular indicator to identify patients at risk of distant metastatic disease. Based on these results, patients with high baseline survivin levels might benefit from intensified chemotherapeutic regimes and may be selected for additional molecular targeted strategies in future clinical trials.

## Competing interests

The authors declare that they have no competing interests.

## Authors’ contributions

IF designed the study, supervised the analysis and prepared the manuscript. CR participated in the design and coordination of the study and revised the manuscript critically. LD provided tumor material and clinical follow up of patients and was involved in the preparation of the manuscript. DK carried out the immunohistochemical analysis and was involved in acquisition of data. MRF participated in the design of the study and was involved in the preparation of the manuscript. SF was involved in immunohistochemical studies and participated in the design and coordination of the study. FR supervised the analysis, was involved in histochemical analysis and contributed substantially in preparing the manuscript. All authors read and approved the final manuscript.
